# Acute alcohol intoxication and the cocktail party problem: do “mocktails” help or hinder?

**DOI:** 10.1007/s00213-021-05924-6

**Published:** 2021-07-27

**Authors:** Alistair J. Harvey, C. Philip Beaman

**Affiliations:** 1 Department of Psychology, University of Portsmouth, King Henry I Street, Portsmouth, PO1 2DY UK; 2 School of Psychology & Clinical Language Sciences, University of Reading, RG6 6AL Reading, UK

**Keywords:** Acute alcohol intoxication, Alcohol myopia, Auditory attention, Selective attention, Working memory capacity, Operation span

## Abstract

**Rationale:**

To test the notion that alcohol impairs auditory attentional control by reducing the listener’s cognitive capacity.

**Objectives:**

We examined the effect of alcohol consumption and working memory span on dichotic speech shadowing and the cocktail party effect—the ability to focus on one of many simultaneous speakers yet still detect mention of one’s name amidst the background speech. Alcohol was expected either to increase name detection, by weakening the inhibition of irrelevant speech, or reduce name detection, by restricting auditory attention on to the primary input channel. Low-span participants were expected to show larger drug impairments than high-span counterparts.

**Methods:**

On completion of the working memory span task, participants (*n* = 81) were randomly assigned to an alcohol or placebo beverage treatment. After alcohol absorption, they shadowed speech presented to one ear while ignoring the synchronised speech of a different speaker presented to the other. Each participant’s first name was covertly embedded in to-be-ignored speech.

**Results:**

The “cocktail party effect” was not affected by alcohol or working memory span, though low-span participants made more shadowing errors and recalled fewer words from the primary channel than high-span counterparts. Bayes factors support a null effect of alcohol on the cocktail party phenomenon, on shadowing errors and on memory for either shadowed or ignored speech.

**Conclusion:**

Findings suggest that an alcoholic beverage producing a moderate level of intoxication (*M* BAC ≈ 0.08%) neither enhances nor impairs the cocktail party effect.

The human ability to comprehend a single speaker amidst a clamour of irrelevant background speech is remarkable. Research into this so-called cocktail party problem began with Cherry’s (1953) dichotic listening task in which participants “shadowed” (i.e. repeated aloud) recorded speech played to one ear (the primary channel) while ignoring an unrelated message simultaneously played to the other ear (the secondary channel). Typically, primary channel shadowing proceeds well but participants usually recall only physical changes to the irrelevant input, such as a switch from male to female speaker, and are oblivious to changes in the meaning of this speech (Cherry 1953).

According to Broadbent’s (1958) classic account, the absence of semantic irrelevant speech analysis implies a blocking filter that controls attentional load by allowing only the target message access to higher processing. For this early selection mechanism to work, ongoing physical analysis of all acoustic inputs must occur for listeners to discriminate primary from non-primary channels. The cocktail party problem is therefore solved by using only physical cues to track the target message, with unattended messages analysed no further. This view was challenged by shadowing studies revealing that around one-third of participants detect their own (but not a control) name in the secondary channel when covertly inserted by researchers, a phenomenon sometimes referred to as the cocktail party effect (Moray 1959; Wood and Cowan 1995). In Wood and Cowan’s (1995) study, own-name detectors showed a temporary drop in shadowing accuracy immediately after name onset, possibly reflecting a shift in attention from the primary to secondary information channel. This led to the notion that Broadbent’s attentional filter must attenuate rather than block non-primary speech inputs (Treisman 1964), although this does not explain why the majority (65%) of Wood and Cowan’s (1995) participants failed to detect their name at all.

Progress on this issue emerged from an individual differences study of speech shadowing by Conway et al. (2001), who observed a significant difference in the name detection rate between listeners with a low (65%) rather than high working memory span (20%)—an effect that has been studied again more recently (Naveh-Benjamin et al. 2014; Röer and Cowan 2021). In reflecting one’s ability to store, manipulate and transform information in real-time, working memory capacity (WMC) is a sensitive measure of cognitive capacity and control (Engle and Hambrick 2016). Conway et al. (2001) suggest that lower-span listeners notice their name because they lack the cognitive resources required to consciously ignore or inhibit the distracting secondary message, as the experimental instructions require. This view receives support from a dichotic listening study by Colflesh and Conway (2007), whose participants shadowed one speech stream while deliberately listening out for their name in secondary speech. Under these instructions, low-WMC participants were less likely to notice their name than high-WMC counterparts, suggesting that the latter group is better equipped to either focus or divide attention in accordance with task demands. From the perspective of Treisman’s (1964) attenuation model, this implies that low-span listeners are less effective than high-spans at damping the irrelevant speech signal, emphasising the importance of working memory capacity for the effective operation of the attenuating filter.

The working memory studies of Conway and colleagues (Conway et al. 2001; Colflesh and Conway 2007) reveal how a particular characteristic can produce differences in auditory attention between individuals, but auditory attention may also be malleable within individuals. For example, relative to unpractised listeners, extended shadowing practice greatly increases the shadower’s ability to spot target digits in the secondary stream (Underwood 1974). It seems likely therefore that the ability to control auditory attention could be moderated within individuals by other means, such as fatigue or drug ingestion. Despite its titular reference to social alcohol consumption, the cocktail party effect has, ironically, been studied amongst only sober listeners. This is surprising because there are good theoretical reasons to expect alcohol consumption to affect auditory attention. Though, interestingly, different perspectives on the influence of alcohol on attention and cognition provide contrasting predictions.

Alcohol has been found to reduce WMC (Colflesh et al. 2010) and increase mind wandering (Sayette et al. 2009), which would presumably increase the likelihood of a cocktail party effect. Further evidence that alcohol restricts cognitive capacity and, therefore, attentional control comes from studies in which intoxicated and sober control groups complete two tasks simultaneously. When attention is divided, the drug typically impairs performance (e.g. slows response time) on one of the tasks (Fillmore and Van Selst 2002). Such attentional limitations may explain the poor self-control of intoxicated individuals, marked by a reduction in the ability to inhibit pre-potent behavioural responses (Fillmore 2007). On this basis, rather like the weaker control associated with low WMC, alcohol should increase the cocktail party effect—reports of noticing one’s own name in a supposedly unattended message.

Alternatively, alcohol may narrow the listener’s focus to the primary channel, reducing the probability of name detection in the secondary channel. An influential review by Steele and Josephs (1990) suggests that alcohol’s depletion of attentional resources reduces peripheral awareness, producing a state of alcohol myopia in which only the most central, goal-relevant inputs are processed. This view is supported by a reaction-time experiment in which acute alcohol produced significantly faster responses to visual targets under auditory interference conditions (deviant novel sounds) relative to placebo controls, suggesting that alcohol helped block the attention-capturing effects of the deviant sounds (Jääskeläinen et al. 1996a, b). In a follow-up forced-choice reaction-time study in which participants had to rapidly categorise the duration of tones, Jääskeläinen, Schröger and Näätänen’s (1999) found participants responded more slowly to surprise changes in tone pitch when sober than when under the influence of just a low dose of alcohol (BAC < 0.04%), again suggesting that the drug limits the ability to involuntarily shift attention to novel stimuli. Low alcohol doses (BACs from 0.04 to 0.06%) also suppress event-related potential (ERP) waveforms. In sober listeners, the N1 and P3a components are associated with an orienting-to-novelty response, as they peak at around 100–250 ms from the onset of a task-irrelevant “oddball” stimulus presented within a repetitive sequence of homogenous tones, but these are significantly reduced following alcohol ingestion (Jääskeläinen et al. 1996a, b; Marinkovic et al. 2001). Importantly, the suppression by alcohol of the change-specific mismatch negativity (MMN) effect has also been observed in a dichotic selective listening scenario (Jääskeläinen et al. 1995). However, the extent to which alcohol’s suppression of attention to surprise deviations in sound frequency may be generalised to the cocktail party effect (e.g. missing one’s own name in the unattended channel) is obviously limited.

The attentional narrowing account of Steele and Josephs (1990) assumes alcohol reduces general cognitive capacity, but Saults et al. (2007) found no evidence of this. In their test of alcohol myopia theory, recall accuracy for stimuli that maximally load working memory—simultaneous auditory or visual arrays—was unaffected by the drug, though intoxicated participants were poorer than controls at recalling auditory and visual sequences. Saults et al. therefore conclude that alcohol disrupts the control processes needed for the rehearsal and maintenance of sequential information but does not shrink overall working memory capacity.

To explore the control limitations alcohol imposes on sequential auditory processing, Fleming et al. (2013) measured ERPs during the discrimination of tones. Alcohol and placebo-control participants listened to a random series of two different tones (350 Hz, 500 Hz) and had to immediately classify each as being either low or high frequency. Sober reaction times slowed when the current tone differed from the two-back tone and this was accompanied by a large P3b amplitude—a standard effect shown in non-pharmacological studies. Under alcohol, however, reaction times slowed when the current tone differed from the one-back tone, with the P3b component now more strongly associated with these one-back changes. This, according to Fleming et al., is evidence that alcohol increases the salience of only the most recently encountered information—a drug effect they refer to as temporal myopia. Though, again, it is unclear whether this suppressive alcohol effect on distinguishing tone sequences extends to the discrimination of verbal information.

In the present study, we therefore replicated Conway et al.'s (2001) design to explore the combined effects of alcohol and cognitive capacity on the cocktail party effect. After completing a working memory span measure, participants were assigned to an alcohol or placebo treatment. Following beverage absorption, they shadowed speech played to one ear while ignoring the voice of a different speaker presented to the other ear. Unbeknownst to participants, their first name had been inserted in the to-be-ignored speech prior to test. Our predictions concerning the influence of alcohol treatment and WMC on this task follow.

If acute alcohol reduces cognitive capacity then it may increase name detection due to a weakened ability to inhibit auditory interference, as shown by Conway et al.’s low working memory span group. Alternatively, alcohol may reduce name noticing by restricting auditory attention to the primary input channel, as suggested by AMT (Steele and Josephs 1990; Jääskeläinen et al. 1995; Jääskeläinen et al. 1996a, b). In line with Conway et al. (2001), we expected more low- than high-WMC individuals to notice their name, though we had no expectations concerning the interaction between alcohol and WMC.

As acute alcohol has been shown to decrease verbal fluency performance (Hartocollis and Johnson, 1956) and suppress verbal fluency practice effects (Benedek and Zöhrer 2020), we expected the alcohol group to make more shadowing errors than sober controls. Conway et al. only examined the effect of working memory capacity on shadowing performance for the word synchronised with the participants own name, plus the two words presented before and after this point. They found no effect of working memory capacity on shadowing errors for the two preceding words, but more errors amongst low-span name-detectors for the name-synced word and the two following it. This presumably reflected a shift in attention from the primary to secondary channel at name onset. We expected to observe a similar difference in shadowing errors between high and low-span participants, again, with no expectations as to how this would interact with alcohol treatment.

We measured the number of words incidentally remembered from the primary channel and expected both low WMC and alcohol to cause lower rates of recall. However, as alcohol may cause a temporal myopia for auditory sequences (Fleming et al. 2013), we predicted a smaller drug deficit for the recall of more recently presented words.

Finally, we expected working memory span and alcohol to influence the incidental recall of secondary channel words other than the participant’s own name. If, as Conway et al. suggest, low-spans experience more secondary channel interference than high-spans, then low-spans ought to remember more words from the “ignored” channel. We were less clear, though, on how alcohol might influence the recall of these secondary channel intrusions.

## Method

### Participants

Eighty-one (51 females, 30 males) undergraduates from the host university with normal hearing were offered either course credit or £10 for participating in the study. The sample ranged in age from 18–60 years (M = 24.70 years; SD = 7.28).

### Apparatus, stimuli and procedure

The experiment was advertised as an investigation of the impact of alcohol intoxication on speech perception. Prior to arriving at the lab, candidates completed an alcohol advisory and screening questionnaire confirming eligibility to participate and urging them not to drive to or from the lab on the day of testing. The screen was designed to exclude respondents under 18 years of age (UK legal age for sale of alcohol), those contraindicated for alcohol on medical grounds and anyone who had not consumed at least eight units of alcohol in a single sitting during the previous 3 months. To screen out alcohol-dependent drinkers, applicants for the study completed a brief version of the Michigan Alcohol Screening Test (MAST; Selzer 1971). Those classified as “problem drinkers” (i.e. a MAST score ≥ 6) were declined from participating. Participants were told not to consume alcohol 24-h before testing and to avoid eating 4-h before testing to reduce breath alcohol (BrAC) variability. All facets of the study were approved by the host university’s ethics committee, and the experiment was administered with full adherence to the British Psychological Society Code of Ethics and Conduct.

Upon arrival for the testing session, each participant was breathalysed to confirm a baseline BrAC of zero and weighed to determine the size of their alcohol dose. Breath alcohol concentrations in participants’ deep lung air were recorded using a Dräger 3000 Alcotest fuel cell breathalyser (mg/100 ml) and converted to BAC estimates based on a 2300:1 blood-breath partition ratio. Prior to beverage administration, participants completed a 5-min random number generation task, the results of which will be reported elsewhere, followed by a measure of working memory capacity known as the operation span (OSPAN) task.

#### Operation span task

We used an automated version of Turner and Engle’s (1989) OSPAN task to measure each participant’s working memory capacity while in a sober state (Unsworth et al. 2005). This computerised test begins with a three-part practice session beginning with an immediate serial recall task. A short sequence of letters appears onscreen for 800 ms, which participants are then required to recall in that order, using mouse clicks to select each letter from a 4 × 3 array (F, H, J, K, L, N, P, Q, R, S, T and Y). The computer provides feedback on the number of letters correctly recalled. In the second practice phase, participants are given a series of maths operations, each to be solved as quickly as possible. Having attempted an equation (e.g. (6 + 4)/2 = ?), participants click to the next screen to reveal a number (e.g. 5) with a “true” and “false” box underneath. They make a single click on one of these to record their response and accuracy feedback follows. For the final practice session, participants perform the letter recall and maths task combined. First, they are first shown the equation then, following a true or false response, a to-be-recalled letter appears onscreen. The task is time limited with participants given only their average practice maths solution time plus 2.5 SD, to solve each equation. If this elapses without a response, the trial is recorded as an error and the programme transitions to the next equation. Participants complete three practice trials each of set size 2. These are followed by the experimental trials with set sizes ranging from 3 to 7. Participants receive 75 equation/letter combinations in total, three at each of the five set sizes, presented in a randomised order (see Unsworth et al. 2005, for further details). Participants completed the automated OSPAN task in approximately 20–25 min and the programme terminates with the production of five scores: the sum of all perfectly recalled letter sets (“OSPAN score”), the total number of letters recalled in the correct position (“total number correct”), the total number of equation errors (“math errors”), the total number of maths errors attributed to a time-out (“speed errors”) and the total number of math errors attributed to miscalculation (“accuracy errors”).

#### Beverage administration

Participants were randomly assigned to an alcohol or placebo control treatment. Males in the alcohol group received 1.5 ml of vodka (40% alcohol by volume), per kg of body weight, mixed with enough sugar-free Indian tonic water to produce a 440-ml drink. As women tend to show higher blood alcohol concentrations than men following the same dose, the alcohol measure administered to females was reduced by 10% to 1.35 ml of vodka per kg of body weight (Frezza et al. 1990; Mumenthaler et al. 1999). Placebo controls were served 440 ml of Indian tonic water with the entire glass mist-sprayed with 10 pumps of neat vodka to give the drink a strong alcohol odour. Beverages were prepared out of view while the participant was engaged with the OSPAN task. Drinks were consumed within 15 min followed by 30 min of rest for alcohol absorption. Participants then had a mouth rinse with water to remove residual alcohol and gave a second undisclosed BrAC recording. Next, the experimenter requested a subjective rating of intoxication recorded on a 10-point scale (1 = “completely sober”; 10 = “extremely drunk”) then issued the selective listening task instructions.

#### Selective listening task

Our selective listening procedure closely replicated that of Conway et al. (2001). Each participant was tested individually under quiet laboratory conditions. Auditory stimuli were presented through Sennheiser stereo headphones at the same (50%) volume for all participants. As in the Conway et al. study, the shadowed message comprised 330 monosyllabic words recorded in a monotone female voice at the rate of 60 words per minute and lasted 5.5 min. The to-be-ignored message contained 300 monosyllabic words recorded in a monotone male voice and began 30 s after the start of the attended message, allowing for a brief practice period without irrelevant speech distraction. The sound recording and editing software Audacity was used to synchronise the words presented in each channel. The same words were always presented in the same order, except for the first names of the present and immediately preceding participants. These were digitally inserted into the irrelevant message in place of a word after 4 and 5 min of shadowing. Following Conway et al. (2001), the position of the participant’s own name was counterbalanced such that half received it at 4 min of shadowing and half at 5 min. The names were obtained when participants emailed their completed screening forms to the experimenter who then inserted into the stimulus track once an appointment was confirmed.

Participants were instructed to shadow the words spoken by the female voice (the only voice played during the first 30 practice words) and ignore the male presenter’s message played to the other ear (introduced after the 30-word practice period). The laterality of each message was counterbalanced across participants to control for hemispheric differences in speech perception (e.g. Wernicke 1874; Scott et al. 2000). Participants were urged to prioritise accuracy by making as few errors as possible and to continue shadowing until the attended message stopped. The experimenter sat at a separate table in the same room and manually recorded shadowing errors. Each score sheet was later checked for accuracy against a digital audio recording of the participant’s output. After the shadowing task, participants were given a brief four-item questionnaire. The first was a request to write down any of the content they remembered from the shadowed speech. The second invited them to report any content they happened to recall from the message they were instructed to ignore. The third was to report anything unusual they remembered from the speech they were told to ignore. The final question asked if they remembered hearing any names in the irrelevant message and, if so, to write these down. Prior to debrief, participants spent 5 min providing a final RNG measure for the unrelated study mentioned previously.

## Results

### Working memory capacity (operation span task)

Our measure of WMC is the absolute automated OSPAN score, which is the sum of all perfectly recalled sets. For example, if 4 letters in a set size of 4, 5 letters in a set size of 5 and 5 letters in a set size of 6 were recalled, the absolute OSPAN score would be 9 (4 + 5 + 0) (Unsworth et al. 2005). The low-WMC group had a mean absolute OSPAN score of 17.53 (SD = 10.44) and the high-WMC group a mean of 45.88 (SD = 10.89), *t*(79) = 11.96, *p* < 0.001. The mean absolute OSPAN score for those randomly assigned to the alcohol group was 31.98 (SD = 15.53) while the placebo group mean was 31.78 (SD = 19.92), *t*(79) = 0.049, *p* = 0.961.

### Intoxication measures

All participants recorded a BAC of 0.00% on arrival for testing. BAC measures for the placebo condition remained at 0.00% throughout the experiment. Thirty minutes after beverage consumption, the alcohol group’s mean BAC was 0.08% (SD = 0.03)—sufficient to preclude driving in the USA and many European countries, including England and Wales—and approximately 45 min later, during debrief, was 0.07% (SD = 0.02). These values are higher than the BACs shown to reduce ERP amplitudes in the Jääskeläinen et al. (1999) study described above. The mean subjective intoxication rating prior to the start of the selective listening task was 2.0 (SD = 1.3) for the placebo group and 4.7 (SD = 1.2) for the alcohol group. There was a strong positive correlation between the objective (BAC) and subjective intoxication measures, *r* = 0.67, *p* < 0.001.

### The “cocktail party” effect

Overall, 52% of participants (*n* = 42) detected their name in the irrelevant speech input and, as shown in Table 1, this approximately even split between detectors and non-detectors was not influenced by alcohol consumption or working memory capacity. Of the 42 participants who detected their name, two detected the paired name inserted in the irrelevant speech. Two of the 39 non-detectors also correctly noticed the paired name in the non-shadowed channel. A loglinear analysis confirmed that the 2(Alcohol Treatment) × 2(WMC Group) × 2(Name Detection) interaction was statistically non-significant, χ^2^ (1, *N* = 81) = 0.017, *p* = 0.897.^1^ A Bayesian version of the same analysis using JASP software (version 0.13.1; JASP Team 2020) confirmed that there was positive evidence for a null interaction effect, BF_01_ = 3.11. This null effect of WMC was a surprise given the positive results previously reported by Conway et al. (2001) and more recent replications by Naveh-Benjamin et al. (2014) and Röer and Cowan (2021). However, there are differences between our procedure and theirs in categorising participants as “high” or “low” WMC. Most notably, Conway et al. (2001) compared the upper and lower quartiles of a sample of 80 participants tested for WMC whereas we employed a less powerful median split given the primary intent to investigate the introduction of alcohol. Röer and Cowan (2021) likewise compared the upper and lower quartiles of their data and Naveh-Benjamin et al. (2014) similarly compared the top and bottom 30% of participants (*n* = 18 and *n* = 16).

**Table 1:** Percentage (count) of listeners who detected their name in the irrelevant speech channel as a function of alcohol treatment and working memory capacity (WMC)

	Low WMC	High WMC	Overall mean
Conway et al. (2001)	13.7%	41.4%	27.6%
This Study	23.3%	60.8%	42.1%
Röer and Cowan (2021)	46.6%	97.15%	71.88%

Naveh-Benjamin et al. (2014) obtained significant differences in name detection between high- and low-span participants (*p* < 0.05), but a more “extreme” difference was reported between younger and older adults with similar working memory capacities (Naveh-Benjamin et al. 2014, Experiment 1, p. 1542). In contrast, however, Röer and Cowan (2021) ran Bayesian analyses across three different methods of classifying participants as high or low WMC (i.e. OSPAN, running memory span and a combination of the two). Despite larger numbers of low-span participants detecting their own name, the authors found only equivocal support for these effects (to two d.p.s, BFs were 1.02 or lower). As a post hoc check on our own results, we therefore examined whether participants who detected their own name had higher WMCs, this time using only Bayesian analyses, so evidence in either direction (for or against the null hypothesis) was generated. Bayes factors obtained for a Bayesian ANOVA showed positive evidence for the null effect of both noticing one’s own name (BF_excl._ = 6.055) and alcohol (BF_excl._ = 6.097) on WMC, and substantial evidence for no interaction (BF_excl._ = 20.309). Further consideration of the reasons for these contrasting results is postponed until the “Discussion” section.

### Shadowing errors

**Fig. 1:**
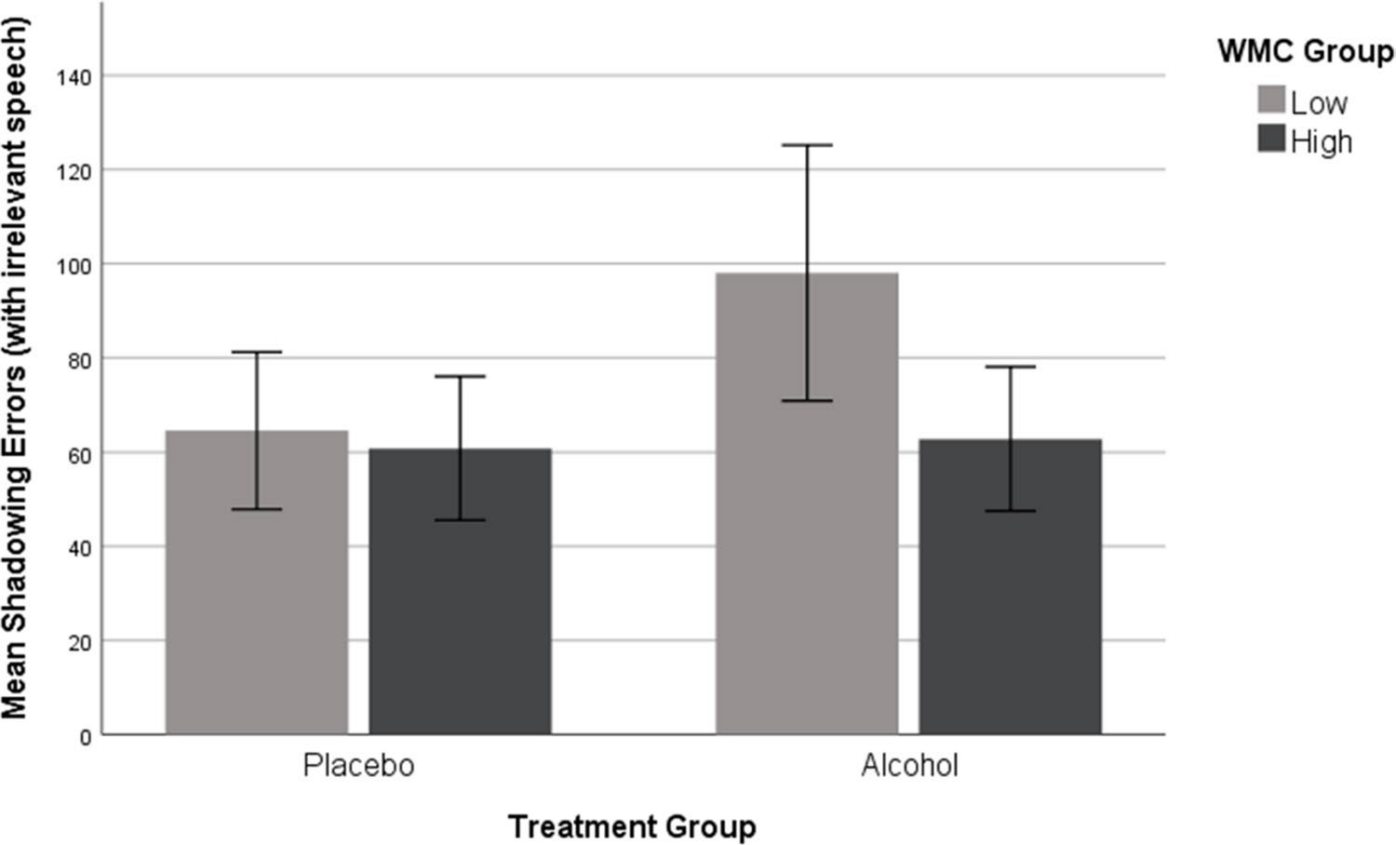
Mean shadowing errors with irrelevant speech as a function of alcohol treatment and working memory capacity. Error bars show 95% confidence intervals

The mean number of shadowing errors made by each group for the 300 shadowed words presented with secondary speech is shown in Fig. 1. We expected alcohol to impair shadowing errors overall, but in an analysis of variance (ANOVA) of square root transformed scores (to homogenise variance), unusually wide variability in the low-WMC group contributed to null effects of alcohol, *F*(1, 77) = 3.499, *p* = 0.065, η_p_^2^ = 0.04, and WMC, *F*(1, 77) = 3.718, *p* = 0.058, η_p_^2^ = 0.05, as well as a non-significant alcohol treatment × WMC group interaction, *F*(1, 77) = 2.087, *p* = 0.153, η_p_^2^ = 0.03. Untransformed scores are displayed in Fig. 1. Bayes factors do little to clarify this situation, as the Bayesian evidence for both main effects and interactions is equivocal (alcohol BF_incl_ = 0.979, WMC BF_incl_ = 1.064, interaction BF_incl_ = 0.719).

**Fig. 2:**
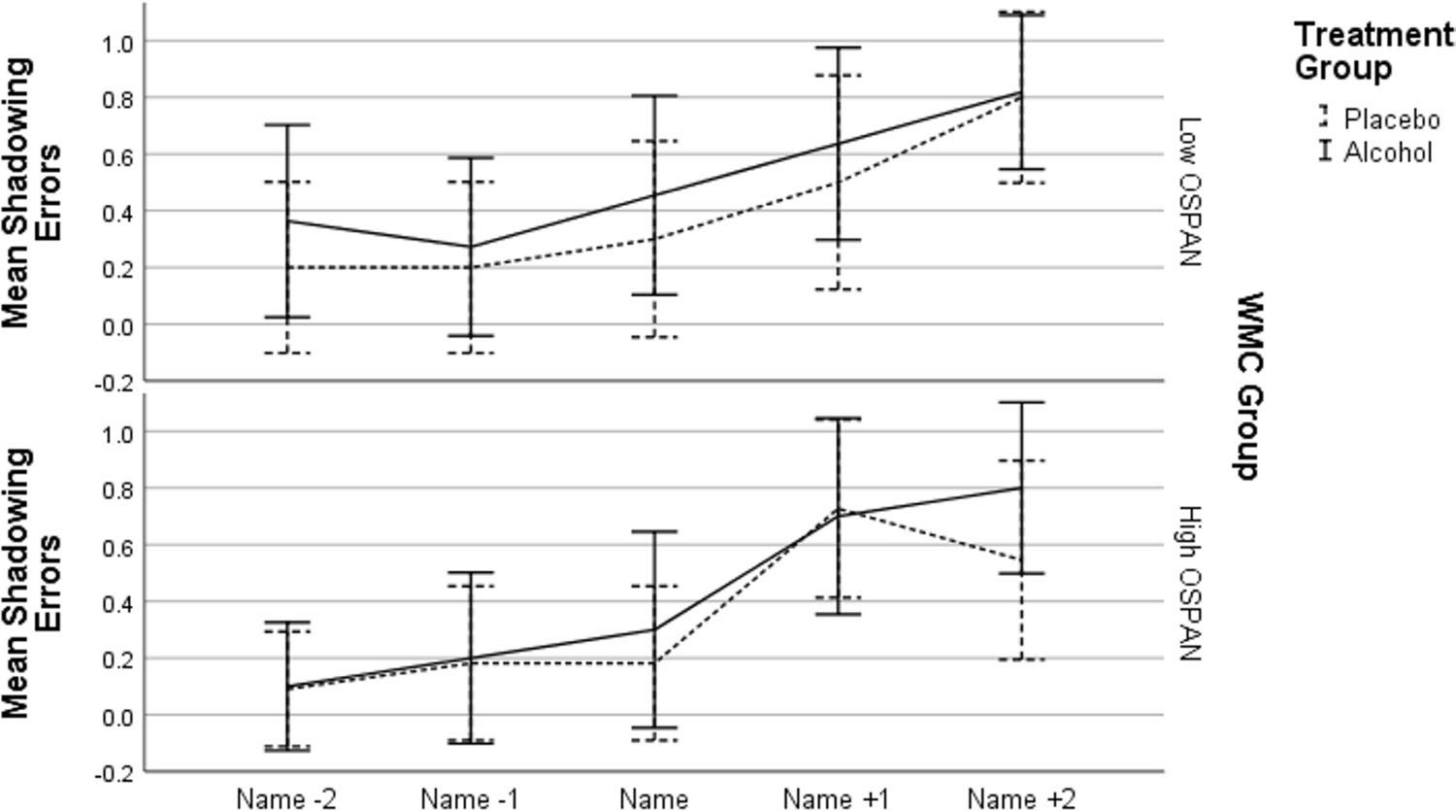
Mean shadowing errors for the name-synced word, the two words preceding it and the two words following it, as a function of alcohol treatment and working memory capacity, for name-detectors only. Error bars show 95% confidence intervals

In the study of Conway et al. (2001), low-WMC participants made significantly more shadowing errors than high-spans right after noticing their name. Figure 2 shows shadowing errors for name-detectors in the present study for the two words prior and after name onset. When we analysed shadowing errors at up to two words before and two words after name presentation, we found a small but statistically significant increase following name onset across all groups, *F*(4, 308) = 14.377, *p* < 0.001, η_p_^2^ = 0.16. Neither the main effects of alcohol and WMC nor their interaction were significant (all *p*s > 0.09). The Bayesian analysis gives similar results—strong evidence for a main effect of when the shadowing error occurred, BF_incl_ = 1.832 × 10^8^, and evidence for the null effect of alcohol, BF_incl_ = 0.209 and WMC, BF_incl_ = 0.112. There was also strong evidence that these factors did not interact as BF_incl_ = 0.042 for the alcohol and WMC interaction, BF_incl_ = 0.02 for the interaction between time/position of the shadowing error and alcohol, BF_incl_ = 0.019 for the interaction between time/position of the shadowing error and WMC and BF_incl_ = 1.593 × 10^−5^ for the three-way interaction. Overall, Röer and Cowan (2021) found that WMC significantly influenced the appearance of shadowing errors (BF = 3.36, 6.49 and 11.68 for each of the three measures of WMC) but not at all word locations, which again contrasts with our results.

### Incidental memory performance

**Fig. 3:**
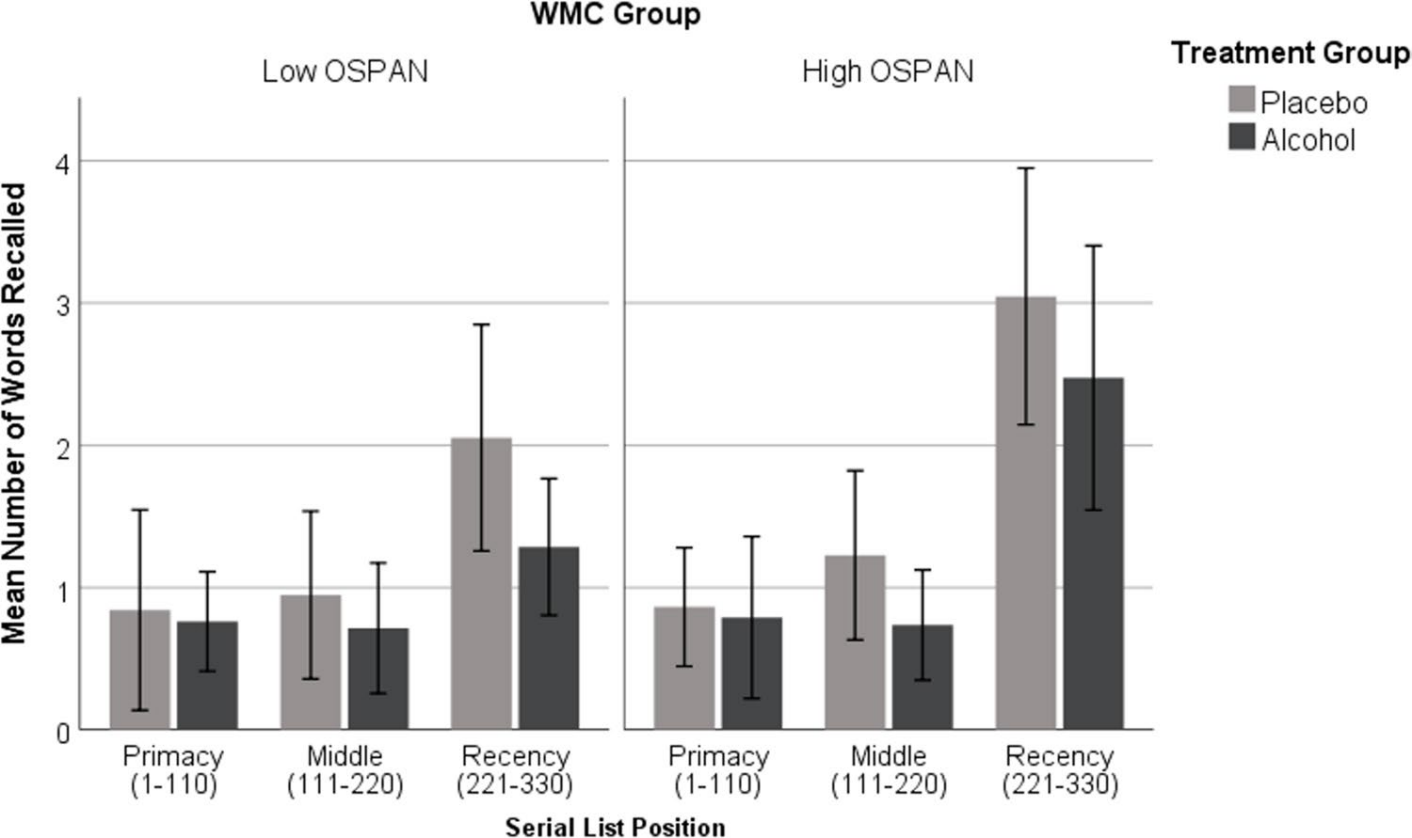
Mean number of primary channel words recalled as a function of alcohol treatment, working memory capacity and list position. Error bars show 95% confidence intervals

The mean number of primary channel words recalled is shown in Fig. 3, as a function of alcohol, WMC and list position. The number of words accurately remembered was low overall, as recall of shadowed words was not an explicit task requirement. Unsurprisingly, only the most recently presented words tended to be remembered, making the main effect of list position significant, *F*(2, 154) = 49.47, *p* < 0.001, η_p_^2^ = 0.34. We expected both alcohol consumption and WMC to reduce the number of words incidentally remembered from the primary channel with an alcohol-induced temporal myopia possibly increasing this deficit (Fleming et al. 2013). There were no significant effects of alcohol on incidental memory, but the interaction between list position and WMC was significant, *F*(2, 154) = 5.405, *p* = 0.007, η_p_^2^ = 0.07, reflecting poorer recall of recency items by low- compared to high-WMC participants. The main effect of WMC just missed significance, *F*(1, 77) = 3.756, *p* = 0.056, η^2^ = 0.02. All other effects were non-significant.

Results of a Bayesian analysis similarly show substantial evidence for an effect of list position BF_incl_ = 9.859 × 10^11^, weak evidence supporting the null hypothesis of no main effect of alcohol, BF_incl_ = 0.428, evidence supporting the interaction between list position and WMC, BF_incl_ = 11.884 and a main effect of WMC, BF_incl_ = 5.05. Null hypotheses were supported for the remaining interactions, but not substantially (BF_incl_ = circa 0.3), except for the three-way interaction where the null result was supported unequivocally, BF_incl_ = 0.055.

**Fig. 4:**
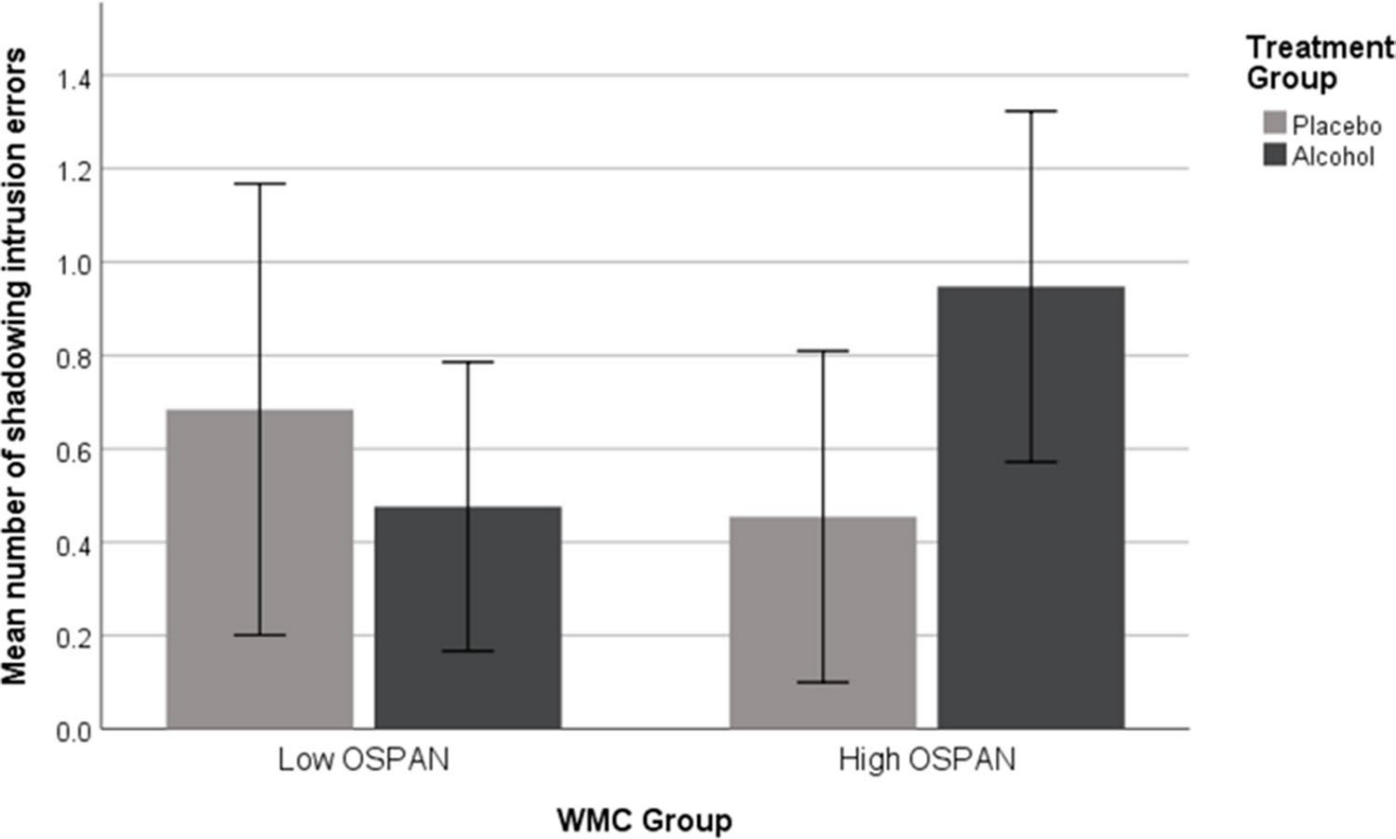
Mean number of shadowing intrusion errors from the secondary channel as a function of alcohol treatment, working memory capacity and list position. Error bars show 95% confidence intervals

Finally, as high-WMC listeners are thought to be superior at inhibiting auditory interference, we expected low-WMC participants to notice and thus recall more words from the “unattended” speech than high-span counterparts. Given the contradictory theories concerning the effect of alcohol on this measure, we made no predictions concerning this variable. The mean number of items recalled by each group is shown in Fig. 4.

We found no significant main effect of WMC, *F*(1, 77) = 0.294, *p* = 0.44, η_p_^2^ = 0.006, or alcohol, *F*(1, 77) = 0.438, *p* = 0.51, η_p_^2^ = 0.008, on the recall of secondary speech items. The WMC × alcohol treatment interaction was also non-significant, *F*(1, 77) = 3.69, *p* = 0.06, η_p_^2^ = 0.046. Bayes factors support these null results, BF_incl_ = 0.233 for WMC, BF_incl_ = 0.252 for alcohol and BF_incl_ = 0.248 for the interaction between these two factors.

## Discussion

The purpose of this investigation was to examine the impact of alcohol on auditory selective attention and, through inclusion of an OSPAN task, the possibility it impairs attentional control by reducing WMC. Like Röer and Cowan (2021), we were unable to replicate the specific finding that, on post-test questioning, more low-span participants reported noticing their own name than high-span participants, which is the measure most usually associated with a “cocktail party effect”, although not the only measure considered by those authors. Bayes factors indicate that the null hypothesis is to be preferred in our study. We also observed a larger overall rate of own-name detection (≈50%) compared to a rate of around 30% shown in most studies (e.g. Conway et al. 2001; Moray 1959; Röer and Cowan 2021; Wood and Cowan 1995), although consistent with the higher rate of 58% younger adults (range 18–21) reported by Naveh-Benjamin et al. (2014).

There are several possible reasons for this discrepancy between our results and those of Conway et al. (2001). In our study, we counterbalanced the ear to which the primary channel was played between left and right whereas all participants in the earlier studies shadowed from the right ear only, a lateral hemispheric bias which may facilitate attentional focus on the primary channel. Another possibility is that our participants, for whatever reason, had more prior knowledge of the “cocktail party effect” than those of Conway et al. (2001) with some, at least, not complying with task instructions by actively listening for targets on the unattended (second) channel. Colflesh and Conway (2007) found that low-span participants were less likely than high-spans to detect their name when expecting it on the second channel. This change in experimental instructions resulted in the opposite pattern to that found by Conway et al. (2001), whose participants were required to ignore the second channel. Naveh-Benjamin et al. (2014, experiment 3), also observed this “opposite” pattern when experimental instructions were switched from ignoring to monitoring a second channel, although the difference in that study was not statistically reliable. A mix of strategies amongst our participants could therefore have cancelled out both effects, though we have no a priori reason for expecting this to be the case. If some participants were actively monitoring the nominally unattended channel then we would anticipate an increase in shadowing errors as a cost of dividing attention, but there is no indication of any interaction between WMC and shadowing errors, although we must stress that the Bayes factor analysis is equivocal as to whether to accept the null hypothesis here, and an overall effect of WMC on shadowing errors was reported by Röer and Cowan (2021) alongside the equivocal results they found for the effect of WMC noticing one’s own name.

A further possibility is raised by the different profile of WMC scores reported between Conway et al.’s (2001) participants and our own. We used a computerised version of the OSPAN test (Unsworth et al. 2005), as did Röer and Cowan (2021), but this differs from Turner and Engle’s (1989) original task as used by Conway et al. (2001). In the Unsworth et al. computerised version, the equations must be recognised as true/false rather than solved, and the to-be-remembered items are not words to be recalled but merely letters to be recognised from an array. Thus, there may be differences between the instruments used even though they are intended to measure the same thing (and typically do so, Conway et al. 2005; Redick et al. 2012). This possibility also lacks support from the results of Röer and Cowan (2021), who found the same patterns in their data regardless of the measurements employed (running span and a combined running span/OSPAN score as well as OSPAN alone). More pertinently, when converted into percentage terms for comparison purposes, our OSPAN results were noticeably higher than those of Conway et al. (2001)—as were those of Röer and Cowan (2021), who obtained only equivocal results with respect to own-name detection (see Table 2).

**Table 2:** Mean OSPAN scores (%) for the low and high-WMC groups from the Cowan and Bunting (2001), Röer and Cowan (2021) and the present study

Treatment group	Alcohol		Placebo	
	Low WMC	High WMC	Low WMC	High WMC
Own name detected	52.4 (11/21)	52.6 (10/19)	47.4 (09/19)	50.0 (11/22)
Own name not detected	47.6 (10/21)	47.4 (09/19)	52.6 (10/19)	50.0 (11/22)

We also used a median split to divide participants into low/high-WMC groups, whereas Conway et al. recruited only the top and bottom quartile from a larger sample of participants who had previously completed the OSPAN test. Median split is a less desirable manipulation than separating groups more clearly, as Conway et al. (2001) achieved, but was employed here because the primary alcohol-based investigation also required a between-participants design. Nevertheless, as Table 2 shows, the mean difference in OSPAN scores between low and high-WMC groups is larger in our study (≈38%) than in Conway et al.’s (≈28%). Although this does not speak to the spread of scores in each of the groups, our Bayesian analysis of differences in WMC (as measured by OSPAN scores) between name noticers and non-noticers provides positive support for the null hypothesis, so we are satisfied that this was not an issue.

A more interesting possibility was raised by careful examination of both OSPAN scores and the percentage of people who noticed their own names. As shown in Table 1, the number of participants who reported noticing their name in the unattended channel was approximately 50% regardless of condition, which is higher than reported in previous studies (e.g. Moray 1959; Wood and Cowan 1995). It is therefore possible that any relationship between WMC and the cocktail party effect is limited to conditions in which one’s own name is less likely to be noticed overall. However, we also note that Naveh-Benjamin et al. (2014) found that the effects of age far outweighed the effects of WMC—in their study younger adults were far more likely to notice their own name than older adults of equivalent WMC, with only 3% of older adults (mean age = 72.5) likely to notice their own name. Age was only controlled for in the Naveh-Benjamin et al. (2014) study, but post hoc examination shows that age ranges were comparable for both WMC groups in our study (mean low-span age = 26.23, mean high-span age = 23.22) and that of Röer and Cowan (2021; mean low-span age = 23.32, mean high-span age = 25.84).

As expected, low-span participants recalled fewer words from the primary channel than high-spans. This effect was statistically significant but only for words in the last third of the list. However, recall of pre-recency items was close to floor so finding that the WMC effect was restricted to “recency” items was not a surprise. Similarly, no effect of WMC was observed on recall of items from the nominally unattended speech input, which was also unsurprising because participants were instructed to ignore this channel. Contrary to the possibility raised earlier, this reassures us that participants were following the instruction to ignore the information presented in the secondary channel.

Although our prediction that alcohol would impair overall shadowing errors was not borne out, we note with interest that low-span listeners who consumed alcohol prior to shadowing made more errors than high-span alcohol counterparts (see Fig. 1). While the overall interaction between alcohol treatment and WMC group was non-significant and we planned no a priori comparisons between the error rates of low and high-WMC intoxicated listeners, we believe this simple main effect warrants exploration in future studies. If alcohol does diminish cognitive resources then low-WMC drinkers may be expected to show a larger shadowing error rate than high-span drinkers, as low-span listeners have fewer reserves to begin with. We therefore speculate that high-span listeners may be better placed to sustain superior shadowing performance in the face of an alcohol challenge.

In terms of the primary motivation for this study, there was positive Bayesian evidence that alcohol—at this dose—neither reduced nor increased the rate at which an individual noticed their own name embedded within a nominally unattended channel. Our findings are therefore inconsistent with the view that alcohol weakens task control (Fillmore and Van Selst 2002; Fillmore 2007) and enhances mind wandering (Sayette et al. 2009), thus increasing the extent to which non-primary information channels are monitored. But nor do they support the attentional narrowing account of Steele and Josephs’ (1990), in which dwindling cognitive resources under alcohol are prioritised for primary information processing, leaving few in reserve for the monitoring and detection of secondary stimuli. This is supported by the work of Jääskeläinen et al. (1995, 1996a, b, 1999) who found evidence of alcohol suppressing attentional shifts to unexpected frequency changes in sequences of distracting tones. That we failed to observe the same effect using more ecologically relevant speech stimuli possibly reflects important differences in the processing of tones and words. Indeed, following an extensive review of non-speech auditory perception stimuli, Schutz and Gillard (2020) point to several instances where the disproportionate use of simplistic tone stimuli in this research field has produced results that fail to generalise to everyday listening scenarios, informing theories that likely underestimate the capabilities of the human auditory system.

It seems a priori unlikely that the present null effects of alcohol would continue at higher doses, but our intervention group’s BAC was at a level which nevertheless precludes driving or operating heavy machinery in many countries, and administering higher doses raises ethical concerns. For many practical purposes, it therefore appears that acute alcohol has no effect on selective auditory attention. In moderation, an alcoholic beverage should render the cocktail party problem neither easier nor more difficult. As such, mocktails should provide no listening benefit for the party-goer relative to his or her mildly intoxicated companion. Higher doses of alcohol may either defocus or narrow attention, as previously discussed, such that more intoxicated individuals may be more or less likely to react to their own name across a crowded party situation. We also cannot rule out the possibility of a dose-dependent effect such that the highest doses have the opposite effect (e.g. an increase in noticing significant information in an “unattended” channel—attentional defocusing) to lower doses (e.g. a decrease in noticing significant information in an “unattended” channel—attentional narrowing). Importantly though, individual differences in WMC did not moderate the effect of alcohol, which suggests the drug will have a similarly null effect on other cognitive control mechanisms associated with working memory capacity, at least at the dose we administered.

## Data Availability

The data that support the findings of this study are openly available in an Open Science Framework repository at https://osf.io/mkxh2/.

## Footnotes

1 Note, the interaction remains non-significant when ear shadowed (left or right) is included in the loglinear model, ^2^ (1, *N* = 81) = 1.459, *p* = .228. A logistic regression further confirmed that neither BAC (β = 5.678, SE = 7.006, *p* = .418), subjective intoxication rating (β =  − 0.131, SE = 0.166, *p* = .428) or WMC (β =  − 0.002, SE = 0.013, *p* = .869) significantly predicted name detection success.
